# Food insecurity in Piura, Peru, in the context of post-earthquake and the COVID-19 pandemic

**DOI:** 10.3389/fpubh.2023.1142550

**Published:** 2023-07-17

**Authors:** Mario J. Valladares-Garrido, Luis E. Zapata-Castro, Abigaíl García-Vicente, Darwin A. León-Figueroa, Yanela Huamani-Colquichagua, Mariana Huaman-Garcia, Raul E. Calle-Preciado, Danai Valladares-Garrido, Cristian Díaz-Vélez, Virgilio E. Failoc-Rojas, Víctor J. Vera-Ponce, César Johan Pereira-Victorio

**Affiliations:** ^1^Escuela de Medicina, Universidad Cesar Vallejo, Piura, Peru; ^2^Oficina de Epidemiología, Hospital Regional Lambayeque, Chiclayo, Peru; ^3^Faculty of Medicine, Universidad Nacional de Piura, Piura, Peru; ^4^Emerge, Unidad de Investigación en Enfermedades Emergentes y Cambio Climático, Lima, Peru; ^5^Faculty of Medicine, Universidad de San Martín de Porres, Chiclayo, Peru; ^6^Facultad de Medicina Hipólito Unanue, Universidad Nacional Federico Villarreal, Lima, Peru; ^7^Unidad de Epidemiología y Salud Ambiental, Hospital de Apoyo II Santa Rosa, Piura, Peru; ^8^Faculty of Medicine, Universidad Privada Antenor Orrego, Trujillo, Peru; ^9^Red Peruana de Salud Colectiva, Lima, Peru; ^10^Unidad de investigación para la generación y síntesis de evidencias en salud, Universidad San Ignacio de Loyola, Lima, Peru; ^11^Instituto de Investigación en Ciencias Biomédicas, Universidad Ricardo Palma, Lima, Peru; ^12^Facultad de Psicologia, Universidad Tecnológica del Perú, Lima, Peru; ^13^School of Medicine, Universidad Continental, Lima, Peru

**Keywords:** earthquake, food supply, food insecurity, COVID-19, Peru, poverty, hunger

## Abstract

**Introduction:**

Piura, located in a seismic zone, faces challenges related to food security. The aim of this study was to analyze aspects related to food insecurity in the region following the 6.1-magnitude earthquake that occurred in 2021, within the context of the COVID-19 pandemic.

**Methods:**

A secondary analysis was performed in 177 people exposed to the earthquake in Piura. Food insecurity was assessed with the Household Food Insecurity Access Scale. Its association with insomnia, resilience, anxiety/depressive symptoms, and general variables were determined using generalized linear models.

**Results:**

Food insecurity was observed in 31.1% (95% CI: 24.3–38.4) of participants. This prevalence was higher in people with mild (PR: 1.73, 95% CI: 1.12–2.70) and moderate (PR: 1.55, 95% CI: 1.09–2.22) anxiety symptoms, severe depressive symptoms (PR: 2.74, 95% CI: 1.24–6.03), and previous exposure to the El Niño Phenomenon (PR: 1.72; 95% CI: 1.06–2.81). An income higher than 5000 Peruvian soles (approximately 1300 US dollars) was associated with a lower prevalence of food insecurity (PR: 0.22, 95% CI: 0.13–0.40).

**Conclusions:**

Overall, three out of 10 individuals experienced food insecurity after the 2021 earthquake in Piura. Food insecurity may be aggravated by mental disorders, previous exposure to the El Niño phenomenon, and the COVID-19 pandemic. Our study contributes to the field by exploring a range of variables related to food insecurity in a unique context: post-earthquake and during the COVID-19 health emergency in Peru. These findings enhance our understanding of food insecurity at the regional level and highlight the need for preventive food security programs in seismic events.

## Introduction

The impact of natural disasters on the economy and people's lives is significant, particularly on populations with low economic resources (1). Earthquakes can compromise nutritional health, 31.6% of survivors had a deficient dietary intake 3 years after the 2011 earthquake in Japan (2). In the capital of Haiti, after the earthquake in 2010, a longitudinal study found that 17.2% of the families informed that their sons had starved; 22.5% had skipped a meal; and 22.6% had reduced their food ration (3). Another study, in southeastern Haiti, found that rural-urban migration was associated significantly with higher food security (4). In Ecuador, after the 7.8-magnitude earthquake, it was observed that out of 28 families, all of them were living in food-insecure households (51.9% mild and 33.3% severe) (5). The factors associated with food insecurity in the context of a natural disaster contain failures that can occur in terms of accessibility (economic and physical barriers, ranging from price increase to road damage), availability (failures in food supply and donation), and food acceptability (religious/cultural reasons, medical prescriptions, etc.) (6).

Even though food insecurity and influencing factors have been evidenced (2–5), the impact of mental health outcomes (post-traumatic stress disorder, acute anxiety disorder, and depression), resilience, insomnia, and social restrictions have not widely been assessed, which could have aggravated food insecurity due to the context of the COVID-19 pandemic (7). Additionally, our sample is more equitable in terms of participant gender and young age (2); in addition, it is bigger in comparison with other studies from the region (5). On the other hand, the vulnerability of the populations has been studied with respect to natural disasters; nevertheless, their previous affectation by frequent natural catastrophes has not been taken into account (2–5). El Niño phenomenon is not a non-frequent event in the region of Piura, Peru, and our study could provide more information about the relationship between the incidence of phenomena like this and the impact on the response capacity of families. This could help in the identification of risk groups and the development of adequate strategies.

Additionally, the COVID-19 pandemic has not only exacerbated physical health (8, 9) but also had a significant impact on food security (10, 11). Factors such as socioeconomic (labor insecurity, price increase, global economic recession, etc.) (12) and mental factors (depression, post-traumatic stress disorder, etc.) (7, 13, 14) have increased population vulnerability, which is really serious in those with the lowest socioeconomic status (15, 16). This should generate greater concern in countries such as Peru, which, until June 2018, had 73% of labor informality according to the National Institute of Statistics and Informatics (INEI) (17), the second highest rate in South America (18). This economic and sanitary crisis affects food quality and security, which generates long-term concerns about healthy eating and its repercussions for health. Hence, the identification of risk groups (people with a low socioeconomic level, orphan children, minorities, older adults, etc.) is key (15).

Piura, a region located in the northwest of Peru, near the Pacific coast, is situated in a highly seismic area due to its location in the Ring of Fire (19–21). Throughout its history, this region has witnessed several significant earthquakes that have left a lasting impact on local communities (19–21). One of the most devastating events occurred in 1912 when Piura experienced the strongest earthquake recorded to date (19–21). This earthquake reached an intensity of 10–11 on the Richter scale, causing an estimated economic loss of one and a half million Peruvian soles in the city of Piura. Moreover, the catastrophe resulted in an uncountable number of fatalities and injuries (19–21).

During the COVID-19 pandemic, the Piura region faced enormous challenges in its healthcare system, resulting in damage to the physical and mental health of its population (22–25). There were errors in containment strategies, oxygen shortages, corruption, and insufficient vaccine distribution. These factors contributed to a difficult situation in the region. This resulted in an epidemiological indicator of a 7.3% fatality rate, ranking it sixth among regions with the highest rate in Peru. According to the Center for Disease Control and Prevention (CDC) of Peru, a total of 13,239 deaths and 181,602 confirmed cases were reported (26). Local businesses and industries, such as tourism and agriculture, suffered significant setbacks, leading to job losses and economic hardships for many residents. Additionally, the education sector faced significant challenges with school closures and the need to implement distance learning solutions (27).

In this context, the earthquake in Piura, a department in northern Peru, occurred on July 30, 2021, probably exacerbated existing food safety challenges in a population already heavily impacted (physically, psychologically, economically, and socio-demographically) by more than a year of the COVID-19 pandemic (7, 10, 28). Therefore, the present study aims to determine the factors associated with food insecurity after the 6.1-magnitude earthquake in Piura in the context of COVID-19.

## Materials and methods

### Study design

We undertook an analytical cross-sectional study with a secondary analysis of databases of the population exposed to the strong 6.1-magnitude earthquake, that occurred on July 30, 2021, in Piura-Peru. The survey was administered in August–September 2021, to determine the prevalence and factors associated with food insecurity.

Piura is a region located in the northwestern zone of Peru, along the country's Pacific coast. It has 8 provinces and 64 districts, with the city of Piura serving as its capital. It ranks second among the most populated regions in Peru. Piura is renowned for its diverse geography and warm climate. The region's economy is primarily based on agriculture, with fishing and mining also being significant industries in the area (29). Piura is prone to natural disasters such as El Niño, which can cause floods and landslides (30, 31).

### Population and sample

The population of the study included people over 18 years of age who experienced the earthquake that occurred on July 30, 2021, in Piura, Peru. The primary study excluded people who were not living in any of the 38 districts of Piura that were declared an emergency due to the damage caused by the seismic movement (32). In this secondary analysis, we excluded participants who did not fill in the HFIAS questionnaire, which measures food insecurity. To calculate the sample size of the primary study, we performed a probability sample with Epidat, using the expected prevalence of 12%, 95% confidence level, and margin of error of 5%, in addition to a 10% rejection rate. Finally, we obtained 179 participants as the final sample. In this analysis, the sample was composed of 177 participants. The primary study used non-probability convenience sampling.

### Procedures of the study

We designed a virtual survey using REDCap, a system of data entry with rigorous properties of quality data collection and control. We disseminated the virtual survey through social networks of health institutions, universities, and media of Piura (WhatsApp, Instagram, and Facebook) to measure the outcomes and exposure of interest in residents of Piura that experienced the strong 6.1-magnitude earthquake that occurred on July 30, 2021, with the epicenter in the west of Sullana, Piura-Peru. The survey was disseminated for 2 months (August and September 2021). To engage the participants' interest, we created a Facebook page to share infographics inviting them to participate in the research through a link that directed them to the REDCap questionnaire.

### Instruments and variables

#### Outcome

##### Household food insecurity access scale

This questionnaire has nine items on the Likert scale from 1 to 3. It has three areas which involve anxiety and uncertainty associated with food supply in the household, food quality, and sufficient food intake and physical effects (33). According to the FANTA-III (Food and Nutrition Technical Assistance) evaluation criteria, a score of mild food insecurity (FI) is given as follows: 2–3 points in the first item, 1–3 in the second item, or 1 in the third or fourth item (33). Moderate FI's scoring is as follows: from 2 to 3 in the third or fourth item, or from 1 to 2 in the fifth or sixth item. In regard to severe FI, it has the following scoring: 3 points in the fifth or sixth item, or 1 out of three in Item 7, Item 8, or Item 9 (33). This scale has been validated for Spanish-speaking Latin populations (33). In this study, we estimated a high internal consistency (total Cronbach's alpha: 0.94 and Cronbach's alpha for each item higher than 0.93) in the evaluated participants. We used this instrument to measure food insecurity in the context of the COVID-19 pandemic in Latin America (34–36).

#### Exposures

##### Generalized anxiety disorder assessment

This questionnaire contains seven items on a Likert scale from 0 to 3 to evaluate anxiety symptoms during the previous 2 weeks of administration (37). It has been validated in Spanish-speaking populations with Cronbach's alpha coefficient of 0.94 (38). Using 10 points as the minimum, sensitivity is at 97%, specificity is at 100%, positive predictive value >99%, and negative predictive value of 0.833 (39). The absence of anxiety was scored from 0 to 4 points, mild anxiety from 5 to 9 points, moderate anxiety from 10 to 14 points, and severe anxiety from 15 to 21 points (40).

##### Patient health questionnaire of depression

This questionnaire has nine items with a Likert scale from 0 to 3 (41). It was validated in the primary care of Hispanics, showing optimal psychometric properties with Cronbach's alpha higher than 0.80, sensibility at 83%, and specificity at 82%, taking a higher than or equal to seven scoring as a cutoff point (42, 43). In addition, it was confirmed in the Peruvian population (*n* = 30.449) by analyzing secondary data from the ENDES survey, 2016, where we found a useful variable for group comparison (42). It is classified into mild depression with 0–4 points, moderate depression with 5–9 points, moderate depression with 10–14 points, moderate–severe depression with 15–19 points, and severe depression with 20–27 points (41).

##### Insomnia severity index

ISI is a self-administered questionnaire that assesses the nature, severity, and impact of insomnia (44). It has seven items with a Likert scale from 0 to 4 (45). The first three elements refer to problems of sleep, sleep maintenance, and early morning awakening problems (46). The four last elements ask about satisfaction with current sleep, others' perception of the participant's current sleep problem, concern about sleep, and interference of sleep problems with daily functioning (46). It has a score from 0 to 28 points, which is interpreted as follows: insomnia absence (0–7), subthreshold insomnia (8–14), moderate insomnia (15–21), and severe insomnia (22–28). It has an acceptable internal consistency of 0.7 (45). ISI has been used in the general Hispanic community (47, 48).

##### Connor-davidson resilience scale

This questionnaire has nine items with a Likert scale from 0 to 4 (49). It has been validated in Hispanic health workers, different workers in various occupational fields, and Hispanic youth (50–53). By having optimal psychometric characteristics, Cronbach's alpha coefficient was higher than 0.80, sensitivity was at 70%, and specificity was at 68.2% to discriminate among health workers, using the cutoff point lower than or equal to 23 (49–52).

### Variables

The dependent variable was food insecurity. This variable was operationally defined as the sum of the scores of the HFIAS, in which a lower score shows lower food insecurity. Low food insecurity was defined as having 2–3 points in Question 1, 1–3 points in Question 2, or 1 point in Question 3 or 4. Moderate food insecurity was defined as having 2–3 points in Question 3 or 4, or 1–2 points in Question 5 or 6. Severe food insecurity was identified when a participant obtained 3 points in Question 5 or 6, or 1–2 in Question 7 or 8.

The independent variables were (1) **Resilience**, initially defined as the addition of the scores of the questions of the abbreviated CD-RISC, in which a higher score was translated as higher resilience. Subsequently, it was categorized using a scoring lower or equal to 23 as a cutoff point. (2) **Insomnia**, defined as a higher or equal to 8 points in the sum of the instruments' questions of ISI. (3) **Labor-sociodemographic variables**: age, gender (female-male), civil status (single, married, cohabiting, divorced, separated, and widowed), education level (none, pre-school, primary, secondary, non-university higher, and university higher), type of employment (worker, domestic worker, student, unemployed, and others), monthly household income in Peruvian currency (300–1,000 soles, 1,001–2,000 soles, 2,001–3,000 soles, 3,001–5,000 soles, and 5,001 or more), religion (Catholic, non-Catholic, and none), and the number of household members. (4) **Medical history of individuals or family members**: frequent consumption of alcohol and tobacco, and comorbidities (none, hypertension, diabetes, obesity, and others). (5) **Stress factors before, during, and after the earthquake**: personal and family mental health history, nervous breakdown occurred immediately after the earthquake, physical injury, house damage due to the earthquake (not affected, mild, moderate, and severe), and loss of employment due to the earthquake. (6) **Social support**: report of social and/or material support by relatives, neighbors, friends, religious partners, politicians, government, and non-governmental organizations (NGOs).

### Statistical analysis

We downloaded the database of the REDCap system in Stata format.

The descriptive analysis showed absolute and relative frequencies in the categorical variables. In the numerical variables, we evaluated normal distribution in a graphic and numerical form and, according to this, the best central tendency measure and dispersion were shown.

The bivariate analysis allowed us to research factors associated with food insecurity. In the categorical variables, we used the independence chi-squared test, before the evaluation of the assumption of expected frequencies. In the numerical variables, we evaluated the assumption of normal distribution. According to this, the Student's *t*-test or Mann–Whitney U-test was useful.

In the simple and multiple regression analysis, we evaluated the factors associated with food insecurity through generalized linear models, log-link function, and robust variance, using the district of residence as a cluster. We estimated prevalence ratios (PR) and confidence intervals at 95% (95% CI). In the multiple regression model, we included the variables that were associated with the simple model (*p* < 0.05). The collinearity of factors associated with food insecurity that were included in the multiple regression model was assessed.

In addition to standard regression analyses, we performed multiple comparisons correction using the Bonferroni method. We chose to apply this correction because we were testing multiple independent hypotheses and wished to control the family-wise error rate to avoid Type I errors. This correction method is known to be conservative and adjusts the statistical significance threshold by dividing the original alpha level by the number of comparisons made. This procedure increases the risk of Type II errors, so results should be interpreted considering both statistical and practical significance.

The statistical analysis was performed in Stata 17.0.

### Ethical aspects

The primary study was approved by the Ethics Committee of the Universidad Norbert Wiener. The confidentiality of the participants was maintained, given that the questionnaires were anonymous. The participants gave their informed consent to participate in the research. This secondary data analysis used an anonymous database.

## Results

### Characteristics of participants

We analyzed a sample of 177 participants. The median age was 22, with 56% of the participants being female and 79.1% being single. Most of the participants (63.8%) had completed a university higher education level, and 55.4% were studying at the university. Additionally, 9.8 and 6.3% reported frequent consumption of alcohol and tobacco, respectively. Moreover, 29.1% of the participants reported being affected by the El Niño phenomenon that occurred in Piura in 2017. When considering the earthquake's effects, 19.2% reported mild damage to their homes, 2.3% had experienced job loss due to the earthquake, and 59.3% had social support from relatives. Furthermore, 41% reported a high resilience pattern, and 7.1% were found with moderate clinical insomnia (Table 1).

**Table 1:** Characteristics of participants (*n* = 177).

**Characteristics**	***N* (%)**
Age (years)^*^	22 (20–29)
**Sex**	
Female	98 (56.0)
Male	77 (44.0)
**Single**	
No	37 (20.9)
Yes	140 (79.1)
**Education level**	
Secondary	46 (26.0)
Non-university higher	18 (10.2)
University higher	113 (63.8)
**Student**	
No	79 (44.6)
Yes	98 (55.4)
**Household income in soles**	
300–1,000 soles	28 (15.8)
300–2,000 soles	65 (36.7)
2,001–3,000 soles	22 (12.4)
3,001–5,000 soles	31 (17.5)
5,001 or more	31 (17.5)
**Religion**	
Catholic	143 (80.8)
Non-Catholic	17 (9.6)
None	17 (9.6)
Household members^**^	4.63 ± 1.87
Alcoholism	17 (9.8)
Smoking	11 (6.3)
**Comorbidity**	
No	147 (84.5)
Hypertension	4 (2.3)
Diabetes	2 (1.2)
Obesity	14 (8.1)
Other	7 (4.0)
Affected by El Niño phenomenon	51 (29.1)
Personal mental health history	12 (6.8)
Family mental health history	20 (11.3)
Physical injury caused by the earthquake	5 (2.8)
**Damage to home due to the earthquake**	
Not affected	140 (79.1)
Mild	34 (19.2)
Moderate	2 (1.1)
Serious	1 (0.6)
Job loss due to the earthquake	4 (2.3)
**Insomnia**	
Absence of clinical insomnia	94 (55.6)
Subclinical insomnia	59 (34.9)
Moderate subclinical insomnia	12 (7.1)
Severe subclinical insomnia	4 (2.4)
**Resilience**	
Low	102 (59.0)
High	71 (41.0)
**Food insecurity**	
Absence	122 (68.9)
Mild	29(16.4)
Moderate	10 (5.7)
Severe	16 (9.0)

^*^Median (25–75^th^ percentile).

^**^Mean ± standard deviation.

### Household food insecurity access scale

**Figure 1:**
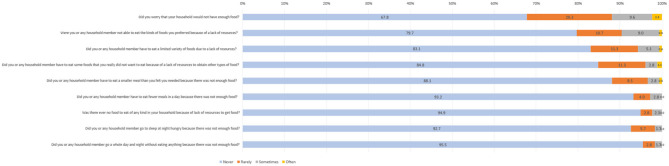
Distribution of responses from the HFIAS questionnaire.

Overall, 31.1% of participants experienced Food Insecurity (FI). Among these, 16.4 and 9.0% exhibited mild and severe FI, respectively. Some of the concerns raised by participants, according to the HFIAS questionnaire, included worries about not having enough food at home and not being able to consume their preferred meals (Table 1; Figure 1).

### Bivariate analysis of factors associated with food insecurity

The bivariate analysis showed statistically significant associations between food insecurity and several factors: age (*p* = 0.020), family income (*p* < 0.001), being affected by the El Niño phenomenon (*p* = 0.009), earthquake's impact on their residence (*p* = 0.001), and the presence of anxiety symptoms (*p* = 0.008) (Table 2).

**Table 2:** Factors associated with food insecurity, in bivariate analysis.

**Variables**	**Food insecurity**		*p* ^*^
	**No (*****n*** = **122)**	**Yes (*****n*** = **55)**	
	***n*** **(%)**	***n*** **(%)**	
Age^**^	21 (20–28)	24 (21–31)	**0.020**
**Sex**			0.058
Female	62 (63.3)	36 (36.7)	
Male	59 (76.6)	18 (23.4)	
Single (yes)	100 (71.4)	40 (28.6)	0.162
**Education level**			0.287
Secondary	30 (65.2)	16 (34.8)	
Non-university higher	10 (55.6)	8 (44.4)	
University higher	82 (72.6)	31 (27.4)	
Student (yes)	75 (76.5)	23 (23.5)	**0.015**
**Household income in soles**			**<0.001**
300–1,000 soles	15 (53.6)	13 (46.4)	
300–2,000 soles	35 (53.9)	30 (46.2)	
2,001–3,000 soles	18 (81.8)	4 (18.2)	
3,001–5,000 soles	26 (83.9)	5 (16.1)	
5,001 or more	28 (90.3)	3 (9.7)	
**Religion**			0.326
Catholic	101 (70.6)	42 (29.4)	
Non-Catholic	9 (52.9)	8 (47.1)	
None	12 (70.6)	5 (29.4)	
Household members^***^	4.79 ± 1.89	4.27 ± 1.78	0.090
Affected by El Niño phenomenon (yes)	28 (54.9)	23 (45.1)	**0.009**
Alcoholism (yes)	10 (58.8)	7(41.2)	0.312
Smoking (yes)	7 (63.6)	4 (36.4)	0.660
Comorbidity (yes)	21 (77.8)	6 (22.2)	0.312
Personal mental health history (yes)	8 (66.7)	4 (33.3)	0.861
Family mental health history (yes)	12 (60.0)	8 (40.0)	0.360
Physical injury caused by the earthquake (yes)	2(40.0)	3 (60.0)	0.156
Damage to home due to the earthquake (yes)	17 (46.0)	20 (54.1)	**0.001**
**Insomnia**			0.201
Absence of clinical insomnia	69 (73.4)	25 (26.6)	
Subclinical insomnia	36 (61.0)	23 (34.0)	
Moderate Subclinical insomnia	9 (75.0)	3 (25.0)	
Severe subclinical insomnia	4 (100.0)	0 (0.0)	
**Resilience**			0.471
Low	68 (66.7)	34 (33.3)	
high	51 (71.8)	20 (28.2)	
**Depression**			0.053
Minimum	67 (78.8)	18 (21.2)	
Mild	32 (64.0)	18 (36.0)	
moderate	14 (56.0)	11 (44.0)	
moderate-severe	4 (44.4)	5 (55.6)	
Severe	4 (57.1)	3 (42.9)	
**Anxiety**			**0.008**
None	68 (80.0)	17 (20.0)	
Mild	35 (61.4)	22 (38.6)	
moderate	13 (48.2)	14 (51.9)	
severe	6 (75.0)	2 (25.0)	

^*^P-value of categorical variables calculated with the chi-squared test.

^**^P-value of categorical-numerical variables calculated with the U test (Mann-Whitney).

^***^P-value of numerical variables calculated with Student's *T*-test with equal variances.

Bold text indicates statistically significant *p*-values.

### Factors associated with food insecurity

**Figure 2:**
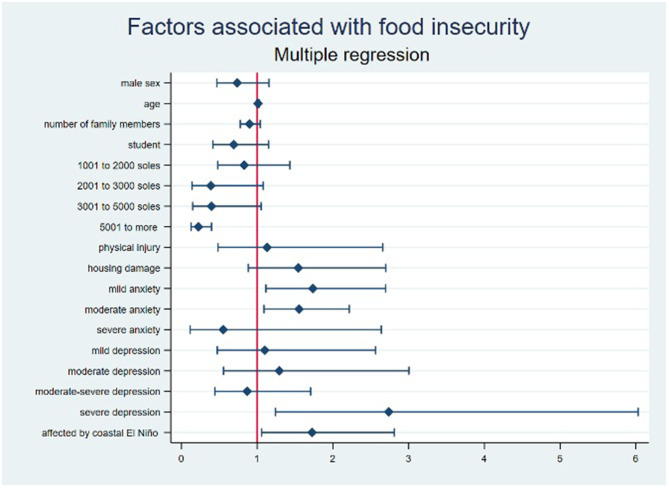
Forest plot of the factors associated with food insecurity in multiple regression analysis.

In the multiple regression analysis, we applied a Bonferroni correction for multiple comparisons to limit the risk of Type I errors, adjusting the significance level to *p* = 0.0025. While severe depressive symptoms (PR: 2.74; 95% CI: 1.24–6.03, *p* = 0.013) and being affected by El Niño phenomenon, 2017 (PR: 1.72; 95% CI: 1.06–2.81, *p* = 0.029), were associated with a higher prevalence of food insecurity, and participants with a family income higher than 5,000 soles had a lower prevalence of food insecurity (PR: 0.22; 95% CI: 0.13–0.40, *p* < 0.001), these factors, except for the high-income group, did not meet the stringent threshold set by the Bonferroni correction (*p* < 0.0025) (see Table 3; Figure 2). Therefore, although these variables were found to be significant under the conventional threshold (*p* < 0.05), they were not significant under the Bonferroni-corrected threshold. This underscores the importance of using conservative methods for assessing statistical significance when conducting multiple comparisons, to minimize the risk of Type I errors.

**Table 3:** Factors associated with food insecurity, in simple and multiple regression analysis.

**Characteristics**	**Simple regression**			**Multiple regression**		
	**PR**	**95% CI**	*p* ^*^	**PR**	**95% CI**	*p* ^*^
Age	1.01	1.00–1.02	**0.013**	1.01	0.98–1.03	0.295
**Sex**						
Female	Ref.			Ref.		
Male	0.64	0.41–0.99	**0.046**	0.74	0.47–1.16	0.182
Single	0.70	0.49–1.02	0.062			
**Education level**						
Secondary	Ref.					
Non-university higher	1.28	0.74–2.22	0.384			
University higher	0.79	0.53–1.17	0.237			
**Student**						
No	Ref.			Ref.		
Yes	0.58	0.42–0.80	**0.001**	0.69	0.41–1.15	0.155
**Household income (Peruvian soles)**						
300–1,000	Ref.			Ref.		
300–2,000	0.99	0.56–1.78	0.984	0.83	0.48–1.43	0.500
2,001–3,000	0.39	0.15–1.01	0.054	0.39	0.14–1.08	0.070
3,001–5,000	0.35	0.13–0.93	**0.035**	0.40	0.15–1.05	0.063
5,001 or more	0.21	0.12–0.37	**0.000**	0.22	0.13–0.40	**<0.001**
**Religion**						
Catholic	Ref.					
Non-Catholic	1.60	0.98–2.62	0.061			
None	1.00	0.46–2.17	0.997			
Household members	0.89	0.80–0.99	**0.037**	0.90	0.78–1.04	0.149
Alcoholism	1.41	0.65–3.05	0.390			
Smoking	1.21	0.66–2.21	0.535			
Comorbidity	0.70	0.45–1.08	0.109			
Personal mental health history	1.08	0.74–1.58	0.696			
Family mental health history	1.34	0.75–2.38	0.327			
Physical injury caused by the earthquake	1.98	1.28–3.08	**0.002**	1.13	0.48–2.66	0.778
Affected by El Niño phenomenon	1.80	1.23–2.66	**0.003**	1.72	1.06–2.81	**0.029**
Damage to home due to the earthquake	2.16	1.52–3.08	**<0.001**	1.54	0.88–2.70	0.129
Insomnia	1.30	0.81–2.10	0.278			
**Resilience**						
Low	Ref.					
High	0.85	0.51–1.40	0.513			
**Depression**						
Minimum	Ref			Ref.		
Mild	1.70	0.88–3.28	0.113	1.10	0.47–2.56	0.823
Moderate	2.08	1.32–3.28	**0.002**	1.29	0.56–3.01	0.553
Moderate-severe	2.62	1.32–5.21	**0.006**	0.87	0.44–1.71	0.682
Severe	2.02	0.95–4.32	0.069	2.74	1.24–6.03	**0.013**
**Anxiety**						
None	Ref.			Ref.		
Mild	1.93	1.16–3.20	**0.011**	1.73	1.12–2.70	**0.014**
Moderate	2.59	1.60–4.21	**<0.001**	1.55	1.09–2.22	**0.015**
Severe	1.25	0.34–4.56	0.735	0.55	0.12–2.64	0.456

^*^P-values obtained with Generalized Linear Models (GLM), Poisson family, log-link function, robust variance, and clustered by districts.

PR, Prevalence ratio; CI, Confidence interval.

Bold text indicates statistically significant *p*-values.

## Discussion

### Prevalence of food insecurity

In our study, we aimed to analyze the prevalence of food insecurity in the context of the earthquake and the COVID-19 pandemic in Piura, Peru. Our findings revealed that approximately one in three people (31.1%) experienced food insecurity following the earthquake, with 9% of the population facing severe food insecurity. Compared to earlier studies conducted in Latin America after natural disasters, these figures are somewhat lower, as previous reports suggested a prevalence of severe food insecurity ranging between 33.3 and 42% (5, 54). Earthquakes generally induce lingering issues such as water pollution, sanitation deficits, and financial difficulties arising from the destruction of rural and agricultural infrastructure (55). Additionally, factors such as overcrowding, increased fertility rates, and expanded household composition contribute to the overall reports of food insecurity (54). Our study acknowledges these underlying issues and their potential influence on the prevalence of food insecurity in the aftermath of the earthquake.

The lower prevalence we observed could be attributed to the timing of our evaluation, 1–2 months post-disaster, and possibly compounded by the concurrent impact of the COVID-19 pandemic (56, 57). The combination of these two crises may have contributed to a complex and multifaceted scenario, where the immediate effects of the earthquake intersected with the pre-existing challenges posed by the pandemic, leading to a unique context of food insecurity.

Given this context, long-term surveillance is crucial to emphasize the importance of long-term surveillance to detect potential increases in food insecurity. The potential impact of socio-economic circumstances and natural disasters on mental health and recovery resilience necessitates particular attention (58, 59). Our study also discovered that worry due to insufficient food is a prevalent indicator of food insecurity, albeit at a lower rate (10%) than in other regions such as India (60) and Ethiopia (61). Moreover, we found that 9% of participants sometimes did not consume the prepared food, reflecting dietary patterns similar to those observed in Japan following the 2011 earthquake (2). This research, conducted in the context of a post-earthquake disaster parallel to a pandemic, underscores the need for comprehensive and sustained monitoring of food security in the region. The study findings highlight the interplay of multiple factors and the potential long-term consequences of these crises on the wellbeing of the affected population. The combined impact of the earthquake and the pandemic has significantly disrupted food availability, access, and the ability of communities to recover.

### Factors influencing food insecurity

Our study discovered that a higher monthly household income corresponded with a reduced prevalence of food insecurity. Income sources and living conditions inversely relate to food insecurity prevalence, a finding aligning with the observations of Hutson et al. (3). Additionally, lack of access to basic services such as electricity, toilets, or running water significantly increased the likelihood of food insecurity post-earthquake. Similar to the observations by Dube et al., we found that economic income impacts food insecurity prevalence, which in turn affects the growth delay in children (62). This association likely arises from the fact that higher income provides improved access to sufficient, safe, and nutritious food, thereby reducing food insecurity (63). In the context of our study, it is essential to understand the relationship between these factors and their implications for post-earthquake and pandemic situations. The combined impact of the earthquake and the ongoing COVID-19 pandemic may have exacerbated existing vulnerabilities and contributed to increased food insecurity. Disruptions in income sources, limited access to essential services, and the overall economic instability caused by these events can have significant implications for individuals' food security. By considering the interconnectedness of income, access to basic services, and the broader context of the earthquake and pandemic, we can gain a more comprehensive understanding of the factors influencing food insecurity. This knowledge is crucial for informing targeted interventions and policy measures aimed at mitigating the effects of these crises and improving food security outcomes in Piura, Peru.

In our study, households that sustained damage exhibited a 70% prevalence of food insecurity. This finding aligns with studies from Peru and Haiti, demonstrating the long-term effects of disasters, like increased delayed growth associated with food insecurity and poverty in severely affected areas (3, 64). Contrastingly, in Nepal, despite significant housing damage post-earthquake, nutritional health remained relatively stable, largely attributed to the implementation of effective rehabilitation and food aid programs (65). The high food insecurity rate in our study might be attributable to the interplay between home damage, household relocation, and economic instability, which are established risk factors for food insecurity during disaster events (66). However, further exploration is needed to determine the exact causes and understand the efficacy of food assistance programs in mitigating food insecurity in the post-earthquake and pandemic context. The long-term effects of the earthquake, such as infrastructure damage, displacement, and economic instability, coupled with the additional challenges posed by the pandemic, likely contribute to the heightened prevalence of food insecurity observed in our study. Future research should continue to monitor and address these issues to develop effective strategies for reducing food insecurity and promoting resilience in similar disaster and pandemic situations.

### Psychological factors and the influence of the El Niño phenomenon on food insecurity

The associations observed in our study among anxiety, depression, and food insecurity warrant careful interpretation. We noted that mild-to-moderate anxiety increased the prevalence of food insecurity, in line with findings from a similar study conducted in Chincha, Peru (67). This echoes evidence from the United States, showing that food insecurity induced by COVID-19 increased the risk of anxiety by 257% among low-income individuals (68). It is crucial to consider regional differences and the co-occurrence of psychological issues in understanding the reciprocal influence of food insecurity and mental health (69). These factors underline the complexity of such relationships and the potential for geographic, cultural, and individual differences in their manifestation.

Our findings of a significant association between severe depression and an increased prevalence of food insecurity align with the study (70, 71). Notably, these associations may be influenced by reduced eating desire or inappropriate eating habits often seen in individuals with severe mental health issues (70, 72). Some studies, however, have reported no significant association between food insecurity and mental health disorders, emphasizing the need for further investigation into the contextual factors that might influence these relationships (73).

Considering the specific context of the post-earthquake and pandemic situation, it is important to recognize that natural disasters and public health emergencies can exacerbate both food insecurity and mental health challenges (74, 75). The disruption caused by the earthquake likely contributed to increased levels of post-traumatic stress disorder (76), anxiety, and depression (77, 78) among the affected population. Moreover, the COVID-19 pandemic introduced additional stressors, such as movement restrictions, economic downturn, and limited access to resources, further compounding the psychological burden experienced by individuals facing food insecurity. Our study findings highlight the interconnections among anxiety, depression, and food insecurity. These associations are influenced by various factors, including the specific context of post-earthquake and pandemic situations.

Participants reported that being affected by the 2017 El Niño phenomenon in Piura showed a significant increase in food insecurity prevalence. This finding parallels research from Haiti showing heightened food insecurity among those severely affected by a hurricane (79), and a study by Edwards et al. associating exposure to disasters and their impacts with severe food insecurity (74). However, these relationships can be complex, potentially influenced by factors like access to economic loans that can mitigate the impact of natural disasters on long-term food insecurity (79).

Given that the El Niño phenomenon is a recurrent event in Piura, and considering the region's location within the Pacific Fire Belt, the population is frequently exposed to disaster risks (80, 81). The cyclical nature of these events may compound the mental health impacts and subsequent food insecurity over time, highlighting the importance of both immediate and long-term support measures. By considering these findings in the context of the earthquake and the ongoing COVID-19 pandemic, it is evident that the combination of these events exacerbates the challenges faced by the population in terms of food security. The post-earthquake situation and the pandemic have likely created additional barriers to accessing food, such as disruptions in the supply chain, economic instability, and limited resources for recovery efforts. Therefore, the observed increase in food insecurity prevalence among those affected by the El Niño phenomenon in Piura serves as an indication of the compounding effects of multiple crises on the local population.

It is essential to note, however, that while severe depressive symptoms and being affected by the 2017 El Niño phenomenon were associated with higher food insecurity at the conventional *p* < 0.05 significance level, they did not meet the more stringent *p* < 0.0025 threshold set by the Bonferroni correction. This underscores the importance of interpreting our findings with caution. The Bonferroni correction, while controlling for Type I errors, might have increased the risk of Type II errors (failing to detect true effects). Therefore, while our results offer valuable insights into the factors influencing food insecurity, the relationships observed should be understood within this context of potential statistical errors. The practical significance of our findings, especially in informing interventions and support programs, is of great importance and warrants further exploration.

### Implications of findings for mental health

This study shows how food security can be impaired by the influence of an earthquake in an environment of socioeconomic inequality. Food insecurity is a complex problem that, if not adequately addressed, can affect the health and quality of life of families in the long term, having a potential impact on the country's economy. The identification of characteristics that increase the frequency of food insecurity, such as those shown in this research, makes it possible to focus interventions on at-risk groups. The findings can contribute to the elaboration of a theoretical framework on the determinants of food insecurity, specifically in the context of a natural disaster. The information provided in this study, together with what was reported in other research, should provide better support for the development of strategies to mitigate food insecurity.

## Limitations and strengths

Interpreting the findings of this study requires mindful consideration of several limitations. First, the cross-sectional study design limits the establishment of causal relationships between the observed variables. As a result, the relationships found are correlational, not causative. Second, potential information bias might exist due to the omission of certain variables, including social support and housing conditions, which could be associated with food insecurity. Third, biases such as recall bias and social desirability bias could affect self-reported variables, possibly leading to misclassification. Fourth, we may have residual confounding as we did not control for specific covariates such as individual vulnerability, social support networks, and community support level. Fifth, the risk of Type 1 errors in our statistical analyses cannot be ignored. Although we have mentioned these risks in the results section and used methods such as Bonferroni correction to control for these, the potential for false positives exists due to the numerous predictors in our models. Importantly, the statistical power of our study was influenced by the relatively small sample size. Although this size was sufficient for detecting typical effect sizes (*r* = 0.20), it had lower power (~25%) for detecting smaller associations (*r* = 0.10) (82, 83). This limitation impairs our ability to identify smaller yet potentially meaningful effects scalable and applicable at the population level (84–86). Future research with larger sample sizes will provide higher statistical power to detect these smaller associations.

In terms of demographic diversity, our sample mostly included individuals in their twenties, thereby limiting the generalizability of our findings. This narrow age range restricts the extrapolation of our results to different age groups that may face unique challenges related to food insecurity. Finally, our use of non-randomized sampling limits the generalizability of our findings to the broader population of Piura. Additionally, while our study used the Household Food Insecurity Access Scale (HFIAS) due to its established use in the literature and its ability to capture different dimensions of food insecurity, we acknowledge that different measures may encapsulate slightly different constructs of food insecurity. Therefore, while our findings may have implications for food insecurity as assessed by other scales (87–89), we caution against directly applying the same associations.

Despite these limitations, our study significantly contributes to the field by exploring a range of variables related to food insecurity in a unique context: post-earthquake and during the COVID-19 health emergency in Peru. This study is among the first to investigate this issue under such conditions, enhancing our understanding of food insecurity at the regional level. Our use of validated measures also adds reliability to our findings. However, future research is needed to address these limitations and further delve into the complex issue of food insecurity in socioeconomically unequal environments and extreme circumstances.

The relationship between the findings of our study on food insecurity and the context of the earthquake and pandemic in the Piura region is crucial for understanding the underlying factors contributing to this issue. It is plausible that the earthquake caused damage to agricultural infrastructure and food availability (55), exacerbating food insecurity in the region. Additionally, the pandemic has generated significant economic impacts, such as job loss and income reduction (90), which may have further worsened the situation. These adverse events, combined with the psychological and emotional stress experienced during these crises, may have created a conducive environment for food insecurity. However, further research is needed to examine in detail the specific interactions among the earthquake, pandemic, and food insecurity, and how these dynamics may vary in different socioeconomic and cultural contexts.

## Conclusion

In conclusion, this study underscores the importance of addressing food insecurity in the unique context of post-earthquake Piura, Peru during the COVID-19 health emergency, a setting already burdened by socioeconomic disparities. We found that household income, house damage, depressive symptomatology, and anxiety symptomatology were significant factors influencing food insecurity. This emphasizes the need for comprehensive interventions, including mental health services and economic support, to alleviate the impacts of food insecurity in similar disaster-stricken areas. Furthermore, longitudinal studies are necessary to validate these findings and to enrich our understanding of food insecurity's determinants and consequences in post-disaster contexts and during global health crises.

## Data availability statement

The data analyzed in this study is subject to the following licenses/restrictions: the Ethics Committee has not provided permission/authorization to publicly share the data but are available from the corresponding author on reasonable request. Requests to access these datasets should be directed to MV-G, mario.valladares@uwiener.edu.pe.

## Ethics statement

The studies involving human participants were reviewed and approved by the Ethics Committee of the Universidad Norbert Wiener, Lima, Peru. The patients/participants provided their written informed consent to participate in this study.

## Author contributions

MV-G: conceptualization, data curation, and methodology. MV-G and LZ-C: formal analysis. LZ-C, AG-V, DL-F, YH-C, MH-G, RC-P, DV-G, CD-V, VF-R, and CP-V: investigation. VF-R, VV-P, and CP-V: supervision. CD-V, VF-R, VV-P, and CP-V: visualization. LZ-C, AG-V, DL-F, YH-C, MH-G, RC-P, VV-P, and DV-G: writing—original draft. MV-G, LZ-C, AG-V, DL-F, YH-C, MH-G, RC-P, DV-G, CD-V, VF-R, VV-P, and CP-V: writing—review and editing. All authors have read and agreed to the published version of the manuscript.
